# Census Bulletin No. 7

**Published:** 1890-09

**Authors:** 


CENSUS BULLETIN No. 7. August 6th, 1890. Robert P. Porter, Superintendent of Census.

This is a periodical which is issued monthly by the Census Bureau in order to bring before the public at as early a date as possible such compilations of the results of the census as have been completed in the short time that has elapsed since the collecting of such a vast mass of data.

The issue now before us relates to the indebtedness of States in 1890 as compared with 1880.

From these returns it appears "that in the decade ending 1890 State indebtedness has decreased in round numbers about $58,000,000."

Census Bulletin No. 8 has also been received. It gives the statistics of the mining of slate. W. M. J.

